# Retraction: Evaluating the spillover effects of the Sugira Muryango home-visiting intervention on temperament of children aged (0.3–3years) exposed to domestic violence: A cluster randomized controlled trial

**DOI:** 10.1371/journal.pone.0327838

**Published:** 2025-07-10

**Authors:** The PLOS One Editors

After publication of this article [1], concerns were raised by author TB that the article [1] includes data published without the owner’s (TB) permission and in the absence of review and approval of [1] by author TB. TB also stated they were not able to verify the analyses because they did not have access to the statistical code used. Furthermore, TB raised concerns about the reported methodology and discrepancies between the sample size, data analysis, and results in [1], and the primary research records. Specifically, they stated that:

- The article [1] states that no power analysis was conducted, however, power calculations were appropriately documented in technical materials pertaining to the study.
- The article [1] reports using the Infant Behavior Questionnaire (IBQ) as the outcome variable for a total sample size of 247 children, but this measure was collected only for a smaller sample size of 57 children ranging from 0 to 12 months of age, and regression analyses were conducted on a smaller subsample of 44 cases.
- Specific selection criteria for the subsample of 44 cases analyzed are not reported in [1].
- The article [1] states that imputations were used in the analysis, but this could not be verified
- Clustering adjustments were not performed in [1].

Author TB therefore stated that the conclusions presented in the article [1] are not supported by the data as analyzed and reported. TB requested retraction of the article.

The corresponding author, TS, stated that there was an error in the reported sample size for the regression analysis, but they consider the conclusions remain supported.

In light of the above concerns pertaining to publication of data without authorization, and the support for the conclusions, the *PLOS One* Editors retract this article.

TB agreed with the retraction. TS and VS did not respond to the final editorial decision. JMN, JM, and SJ either did not respond directly or could not be reached.
